# Rapid adsorption of some heavy metals using extracted chitosan anchored with new aldehyde to form a schiff base

**DOI:** 10.1371/journal.pone.0274123

**Published:** 2022-09-09

**Authors:** Huda Y. Sharef, Nabil A. Fakhre

**Affiliations:** Department of Chemistry, College of Education, Salahuddin University-Erbil, Erbil, Iraq; Universiti Brunei Darussalam, BRUNEI DARUSSALAM

## Abstract

A new aldehyde 2,2’-[propane-1,3-diylbis(oxy)] dibenzaldehyde was synthesized from refluxing 2-hydroxy acetophenone and 2-hydroxy 1,3-dichloropropanean in an alcoholic medium. The compositions and properties of the new aldehyde compound were characterized by elemental analysis, FTIR, and nuclear magnetic resonance spectroscopy studies. The extracted chitosan was made to react with a new aldehyde to form a Schiff base by a suitable method. The effects of initial concentration of metal ions, exposure time, imine weight, and pH on the adsorption of Cu(II), Cr(III), and Zn(II) metal ions were examined. An adsorption batch experiment was conducted. The adsorption process followed a second-order reaction and Langmuir model with qe 25 mg/g, 121 mg/g, and 26.31 mg/g for Cu(II), Zn(II), and Cr(III) respectively. The Gibbs free energy showed a negative value and the adsorption/desorption tests provided a high value 5 times.

## Introduction

Heavy metal accumulation in an ecological community is a worldwide problem. The rising concentration of heavy metals in drinking water poses a major threat to physical fitness and the natural system. Heavy metals are one of the major significant polluters of the environment [1]. Transition metal poisoning from industrial effluent is a serious issue. Many sectors, including electroplate, metallurgical processes, pigments, mining, and the leather industry, emit varying levels of transition metal ions. Zinc, cadmium, chromium, copper, lead, manganese, and iron are common metal ions found in both natural and industrial effluent [2].

A variety of conventional approaches have been used. There is a plethora of options, different methods through which transition metal ions may be removed from wastewater. Some of the most common methods include membrane filtering, electrolysis, ion exchange, activated carbon adsorption, and electrolysis. Chemical precipitation, for example, is another method. Other concerns include secondary contamination; prohibited processing capacity, high cost, poor selectivity, and energy consumption are all its drawbacks [3].

Due to its reliability and complexity, adsorption is the most economical and ecologically beneficial method. Adsorption of heavy metal ions now comprises biosorbents from renewable natural sources and modified forms [4]. To increase the inhibitory activity of chitosan, scientists modified its molecular structure [5]. A process was performed where chitosan amino groups were combined with aldehyde derivatives to produce Schiff base [6].

Aromatic aldehyde derivatives are fundamental intermediates that are widely utilized for the synthesis of important materials [7]. Schiff base are compounds with an azomethine group that arise as the result of a reversible acid-catalyzed condensation reaction between primary amine and carbonyl compounds, as described by Hugo Schiff in 1864 [8].

Gokila S. et al synthesized chitosan and Alginate nanocomposites by an ionic crosslinking method, this new biosorbent can remove Cr ion from waste water [9]. Vivian L. et al synthesized of three different imines, by functionalization of chitosan to form Schiff base using three aldehydes. To uptake some heavy metals from water at basic pH’s [6]. Narjes N. et al, used cationic copolymerization technique to prepare A novel Schiff base on porous chitosan-glutaraldehyde /montmorrilonite nanoparticles modified with 3-aminopropyl triethoxysilane nanocomposite with high thermal stability to remove Pb ion and Hg ion [10]. The magnetically modified chitosan, MCS-PPIMB, was prepared by Shahraki S. et al, using aromatic ring-rich in schiff base ligand. This new adsorbent was suitable candidate for Pb ion adsorption in aqueous environments [11].

It was reported that chitosan Schiff bases have excellent chelation ability with heavy metal ions, because it can act as a good binding site for many transition metals and therefore can form stable coordination complexes [12,13]. In azomethine derivatives, the C = N linkage is essential for biological activity; several azomethines were reported to possess remarkable antibacterial, antifungal, anticancer, and diuretic activities. Schiff bases have wide applications in food industry, dye industry, analytical chemistry, catalysis, fungicidal, agrochemical, and biological activities [2].

The purpose of the study was to increase the importance of the chemical modification of chitosan through its functionalization with new aromatic aldehyde, by condensation of (2-hydroxybenzaldehyde) and (3-dichloropropane) in the ratio (2:1) to get a new schiff base with high adsorption capacity with huge specific surface area, excellent pore morphology, selectivity and recycled and reused numerous times without losses of the adsorption activity. The prepared adsorbent was characterized by Fourier Transform Infrared spectra (FTIR), Field Emission Scanning Electron Microscopy (FESEM), Energy Dispersive Spectroscopy (EDX), X-Ray Diffraction (XRD), and Nuclear magnetic resonance H-NMR. Contact time, starting metal concentration, pH, temperature, adsorbent dosage, and selective adsorption kinetic, thermodynamics, and reuse were examined.

## Material and methods

### Reagents

All reagents used were of analytical reagent grade stated. Chitosan (CS) (with DDA 64% from IR chart) was extracted from shrimp shell were purchased from local market. Sodium hydroxide (NaOH, 97%) was purchased from Scharlau. Hydrochloric acid (HCl, 37%) and Glacial Acetic acid (CH_3_COOH, 99.8%) were purchased from Merck. CuSO_4_.5H_2_O (99%) and ZnSO_4_.7H_2_O (99–100.5) were purchased from Sigma-Aldrich, Merck respectively. Cr_2_(SO_4_)_3_.15H_2_O (99%) and 3-dichloropropane (98%) were purchased from Riedel-De Haen AG. Na_2_CO_3_ (≥ 99.5%) and EDTA (99.4–100.6%) were purchased from BDH. absolute Ethanol and 2-hydroxybenzaldehyde were purchased from Sigma-Aldrich.

### Chitosan extracted from shrimp shell

The conventional techniques were used to complete the chitosan extraction procedure. It included the removal of demineralization, deproteination, and deacetylation. A short while later, the shrimp shells were cleaned, dried, and crushed to make them easier to eat. Then, the crushed shells were dissolved in hot water containing 4% (w/v) sodium hydroxide to dissolve proteins and sugars. It was done using 1% HCl for 24 hours, which demineralized the shells.

Finally, a 50% sodium hydroxide solution was added to the previous combination, then warmed up at 100°C for 2 hours to finish the reaction process. Following a thorough rinse with running tap water and distilled water to neutralize the solution, a vacuum oven set to 60°C was used to dried extracted chitosan [14,15].

### Preparation of 2,2’-[propane-1,3-diylbis(oxy)] dibenzaldehyde

According to the modified method [16], a stirred solution of 2-hydroxybenzaldehyde (9.77 g, 0.08 mol) in 100 ml absolute ethanol was boiled in round bottle flask and stirred with a magnetic stirrer; then slowly, Na_2_CO_3_ (33.92 g, 0.32 mol) and 3-dichloropropane (5.16 g, 0.04 mol) in ethanol (30 ml) was added dropwise. The mixture was refluxed for 8 h at 180–200°C. The flask was put in an ice bath. The precipitate was filtrated, dried, and recrystallized using chloroform/methanol solution (1:1) to give the white product (**yield 85%, 115–117**°**C**).

### Chitosan-Schiff base synthesis (imine)

The methods documented in the literature were used to produce the chitosan-Schiff base [17]. It was synthesized through a condensation process. About 1.0 g of chitosan powder was dissolved in 25 ml ethanol with 3 drops of acetic acid and vigorously shaken to produce an emulsion of chitosan. Additionally, 0.87 g of new aldehyde 2,2’-[propane-1,3-diylbis(oxy)] dibenzaldehyde was dissolved in 25 ml ethanol and added to the Chitosan emulsions. Before heating the contents for 12 h in a 60°C underwater bath, both solutions were combined and agitated for 30 min.

The orange product was filtered and dried after being rinsed with ethanol (2-(3-(2-((E)-(((2R,3R,4R,5S,6S)-4-hydroxy-6-(hydroxymethyl)-2-methoxy-5-methyltetrahydro-2H-pyran-3-yl) imino-methyl-phenoxy-propoxy-benzaldehyde).

### Characterization of imine

The synthesized imine was characterized in a way that covers a large area. Fourier transfer infrared spectra were carried out (Shimadzu IRAffinity–I FTIR spectrophotometer), the morphologies of particles were observed using FESEM coupled with EDX, (TESCAN MIRA3 FEG-SEM, Czech Republic) at 15kV under low vacuum after coating with gold thin film, with SE detector for EDX. and XRD patterns were recorded with an X-ray diffractometer using a Cu Kα spectral line at 45 kV and 40 mA and a 2θ between 5 to 80°. Finally, ^1^H-NMR (broker AVANCENEO (400MHZ) spectrometer).

### Adsorption procedure

The batch experiment was conducted to investigate the impact of dose using various amounts of sorbent—0.01 g, 0.015 g, 0.02 g, 0.025 g, 0.03 g—with pH ranging from 3 to 11, The initial ion concentration for Cu(II) and Cr(III) 5–100 mg/L and10–400 mg/L Zn(II); the contact duration 5–300 min, and temperature 5–45°C. The new adsorbents (0.02 g) were added to 10 ml of a heavy metal aqueous solution, which was then shaken at ambient temperature.

The residual concentration of heavy metal ions after adsorption became determined using a FAAS, and the absorbance at 324.8 nm, 213.9 nm, and 357.9 nm for Cu(II), Zn(II), and Cr(III), was observed respectively with a spectral bandwidth of 0.5 nm. The adsorption capacity utilizing the following Eq (1):

$$qe=\frac{(Ci-Ce)V}{M}$$ (1)

The q_e_ ions have an equilibrium capacity of adsorption, which is described by the constant concentration ratio of adsorbent and the initial concentration of ions. Additionally, C_i_ (mg/L), and C_e_ (mg/L), the initial and final concentrations of metal ions, respectively, are used to indicate the ion concentrations at the start and equilibrium of a reaction. The volume of the ion solution is defined by the measurement V (L), and the mass of the adsorbent is defined by the M (g).

The Langmuir, Freundlich, and Temkin models have been used to estimate the adsorption data for the mechanism of the adsorption process [18]. The Langmuir equation can be written in the linear form Eq (2):

$$\frac{Ce}{qe}=\frac{1}{qmax\;Kʟ}+\frac{Ce}{qmax}$$ (2)

Kʟ is the adsorption-related Langmuir constant (mg/g), and qmax maximum adsorption capacity. Which may be related to changes in the reasonably normality and porosity of the adsorbent that would lead to higher adsorption ability for a bigger surface area and porous volume. In describing the basic characteristics of the Langmuir isotherm, the separation factor R_L_ is a dimensionless constant as illustrated in Eq (3):

$$Rʟ=\frac{1}{1+Kʟ\;Ci}$$ (3)

The separation factor R_L_, a dimensionless constant. The adsorption process unfavorable When R_L_ > 1, linear when R_L_ = 1, favorable when 0 < R_L_ ˃1, and irreversible when R_L_ = 0.

Meanwhile, the Freundlich isotherm has the following linear form as shown in Eq (4):

$$\log\;qe=\log\;Kf+\frac{1}{n}\log\;Ce$$ (4)

K_*f*_ represents adsorption capacity (L/mg) and 1/n denotes adsorption intensity; it also denotes the energy distribution and adsorbate site heterogeneity.

The linear forms of the Temkin isotherm may be expressed by Eq (5):

qe=at+2.303btlogCe (5)

The Temkin constant (bt) is related to the heat of sorption (J/mol) and the Temkin isotherm constant (at) (L/g).

The adsorption behaviour during biosorption was investigated using a pseudo-1^st^ order kinetic model and a pseudo-2^nd^ order kinetic model in this research [19].

Eq 6 is the pseudo-1^st^ order kinetic model:

$$log\;(qe-qt)=\log\;qe-\frac{K_{1}t}{2.303}$$ (6)

The pseudo-2^nd^ order kinetic model as explained by linear form Eq (7):

$$\frac{t}{qt}=\frac{t}{qe}+\frac{1}{K_{2}{qe}^{2}}$$ (7)

where K_1_ is the pseudo-1^st^ order kinetic adsorption rate constant (min^-1^) and K_2_ is the pseudo-2^nd^ order kinetic adsorption rate constant (g/mg min).

### Real sample preparation

The efficiency of the Schiff base was evaluated with a determination of Cu(II), Zn(II), and Cr (III) ions in some supplicates. The study took a sample from the two types of nutritional supplement tablets that bodybuilders take (Heavy metal H.M. and multivitamin M.V.). The heavy metal content was digested using 1:3 HClO_3_ and HNO_3_. Then, 25 ml of deionized water (DW) was added to dilute the sample. 10 ml of diluted sample was transferred into a tube and 0.02 g of adsorbent was placed, then shaken using thermostat water bath shaker at an optimum condition that was optimized previously. The supplement was applied on imine by batch adsorption and recovery tests and FAAS was used to calculate the heavy metal ions ratio.

## Results and discussion

### Aldehyde characterization

The new aldehyde, 2’-[propane-1,3-diylbis(oxy)] dibenzaldehyde was prepared in good yield (85%) based on Williamson ether synthesis between 2-hydroxybenzaldehyde and 1,3 dichloro-2-propanol.



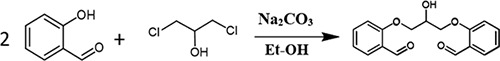



**Fig 1:**
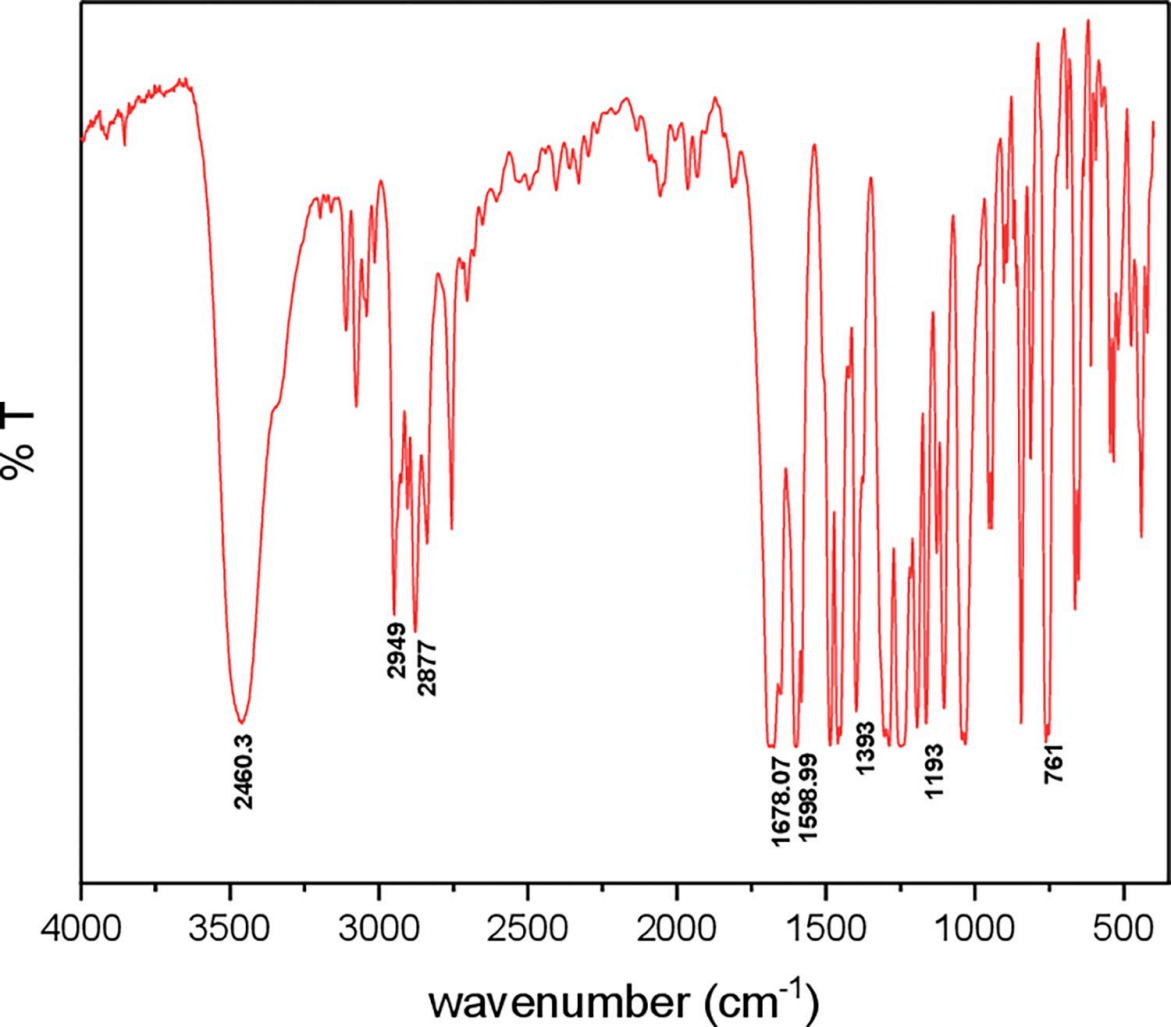
Represents FT-IR spectrum 2,2’-(propane-1,3-diylbis(oxy)) dibenzaldehyde (compound 1).

The structure of the synthesized chemical was determined using FT-IR and ^1^H-NMR techniques, as shown in **Tables 1** and **2** respectively. In the IR spectrum of compound (1) **Fig 1**, the appearance of broadband at 3460.3 cm^-1^ is attributed to the (OH) group, and the strong band at 1678 cm^-1^ refers to the carbonyl group. The ^1^H-NMR spectrum of compound (1) (S1 Fig), as illustrated in **Table 2** shows a signal at 4.26 ppm which refers to the hydroxyl group, while a 4.31 ppm belongs to the methylene proton. A quintet at 4.55 ppm refers to CH- aliphatic protons, a multiplet at 7.0–7.8 ppm corresponds to the eight protons of the two aromatic rings, and a single signal at 10.48 ppm belongs to the proton of an aldehyde group.

**Table 1:** *Shows the FT-IR data of synthesized compounds 1*.

2,2-(propane-1,3-diylbis(oxy)dibenzaldehyde.		
OH str. (cm^-1^)	C = O str. (cm^-1^)	C-H Aliphatic str. (cm^-1^)
3460.3	1678.07	2877 2949

**Table 2:** Shows the H1-NMR data of compound 1.

No	δ/ppm	Multiple.	Intens.	Assign
**1**	4.26	D	1H	-OH
**2**	4.29–4.35	Dd	4H	-OCH_2_-
**3**	4.55	Quintet	1H	-CH-OH
**4**	7.0–7.8	M	8H	Aromatic
**5**	10.48	S	2H	-CHO

### Characterization of imine (Schiff base)

#### FTIR

**Fig 2:**
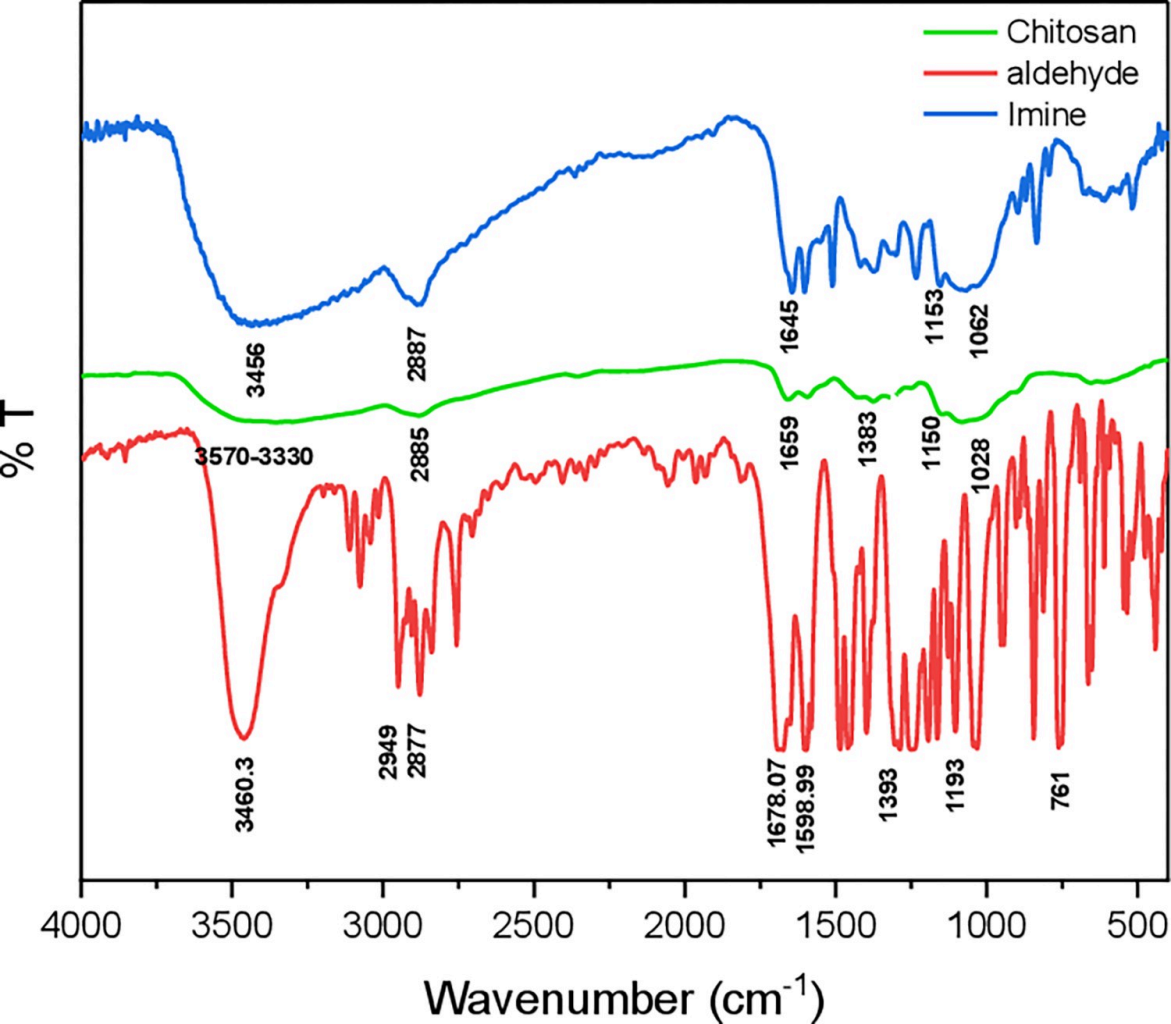
FTIR for Chitosan, Aldehyde (Compound 1), and Imine.

FTIR spectra of chitosan, aromatic aldehyde, and imine are shown in **Fig 2** the stretching vibration of the OH and NH_2_ functional groups is attributed to the wide peak at 3570–3330 cm^-1^, whereas the stretching vibration of the (CH) group of the chitosan backbone is assigned to the height at 2885cm^-1^. Other peaks associated with the amide group include those at 1083 cm^-1^, 1150 cm^-1^, 1028 cm^-1^ (stretching vibration of the C-N bond), 1383 cm^-1^ (stretching vibration of the C-O bond), and the absorption peak at 1659 cm^-1^ [10,20]. The high absorbance band at 1645cm^-1^ for chitosan -imine is due to the C = N vibration, which is typical of the imine produced among the NH_2_ group of chitosan and the carbonyl group of aldehyde [17]. Because the free aldehyde group is condensed along with a primary amine in the basic chitosan monomer and produces imine, no peak was seen between 1720 cm^-1^ and 1740 cm^-1^ and 2947 cm^-1^ and 2877cm^-1^. It means that the C-H stretching of aldehyde bonded with the N-H of chitosan. [11,17].

#### ^1^H-NMR

The H-NMR signal chemical shifts of the studied Schiff base–imine 2-(3-(2-((E)-(((2R,3R,4R,5S,6S)-4-hydroxy-6-(hydroxymethyl)-2-methoxy-5-methyltetrahydro-2H-pyran-3-yl)imino)methyl)phenoxy)propoxy)benzaldehyde recorded in DMSO **(**S2 Fig**)** The spectrum provides the following signals: phenyl as a multiplet at 6.8–8 δ, -N-CH_2_ at 4.5 δ, and C-CH = N- proton at 9.8 ppm. This shifted occurrence in the spectrum on account of the high electronegativity of fluoride in an aromatic ring [21–23].

#### FESEM

**Fig 3:**
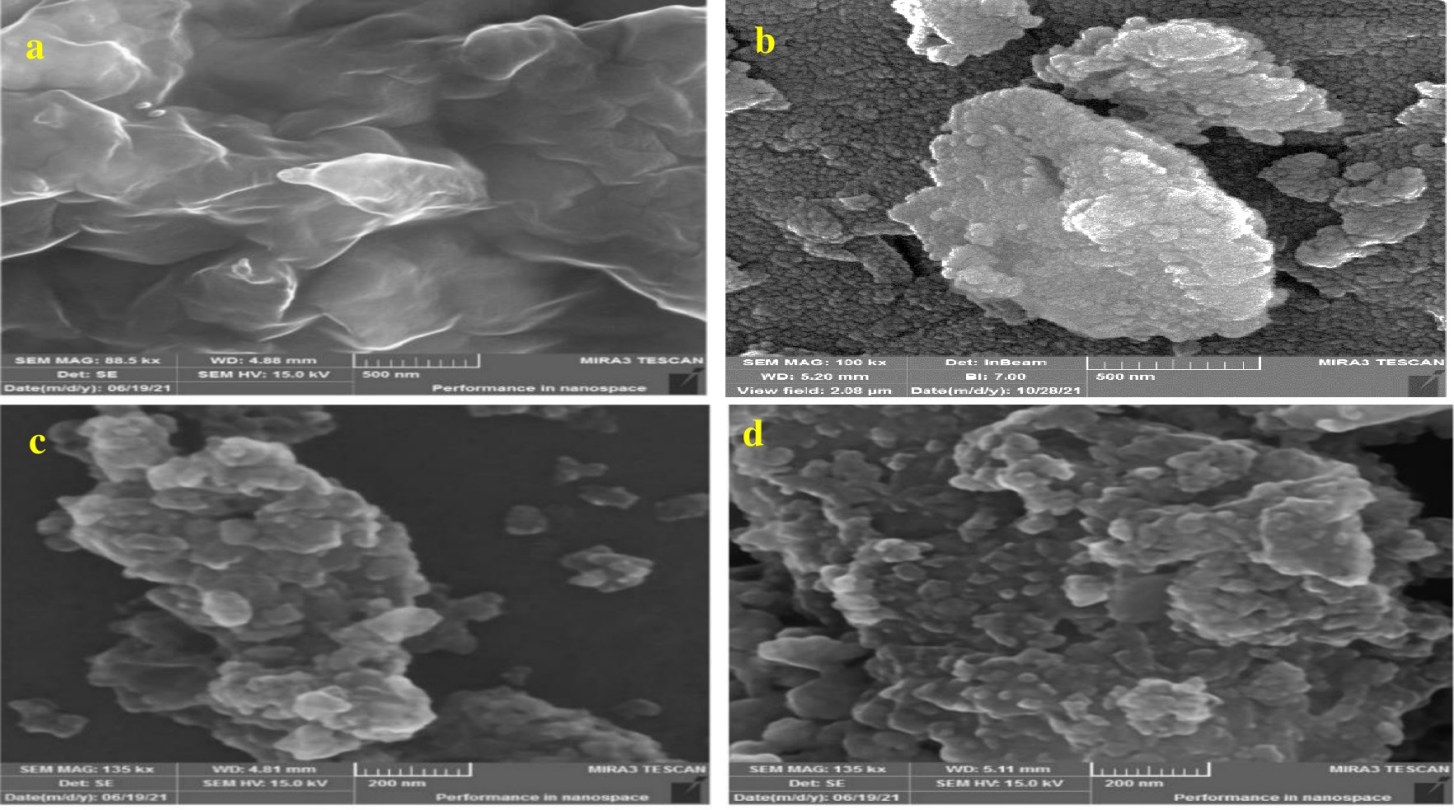
FESEM images of (a) Imine before adsorption (88.5 kx, 500 nm), (b) after adsorption of Cu(II) ion (100 kx, 500 nm), (c) after adsorption of Zn(II) ion, (135 kx, 200 nm) and (d) after adsorption of Cr(III) ion (135 kx, 200 nm).

Sorbent particle geometry before and after adsorption using the FESEM (Field Emission Scanning Electron Microscopy) to clarify the nature of the new imine adsorbent. The adsorbent surface was uneven with various grooves, favourable for adsorption **Fig 3A**. The significant change observed with imine Cu(II), Zn(II), and Cr(III) that had been more rugged with spherical shape particles are recognized on the surface **Fig 3B–3D.**

#### EDX

**Fig 4:**
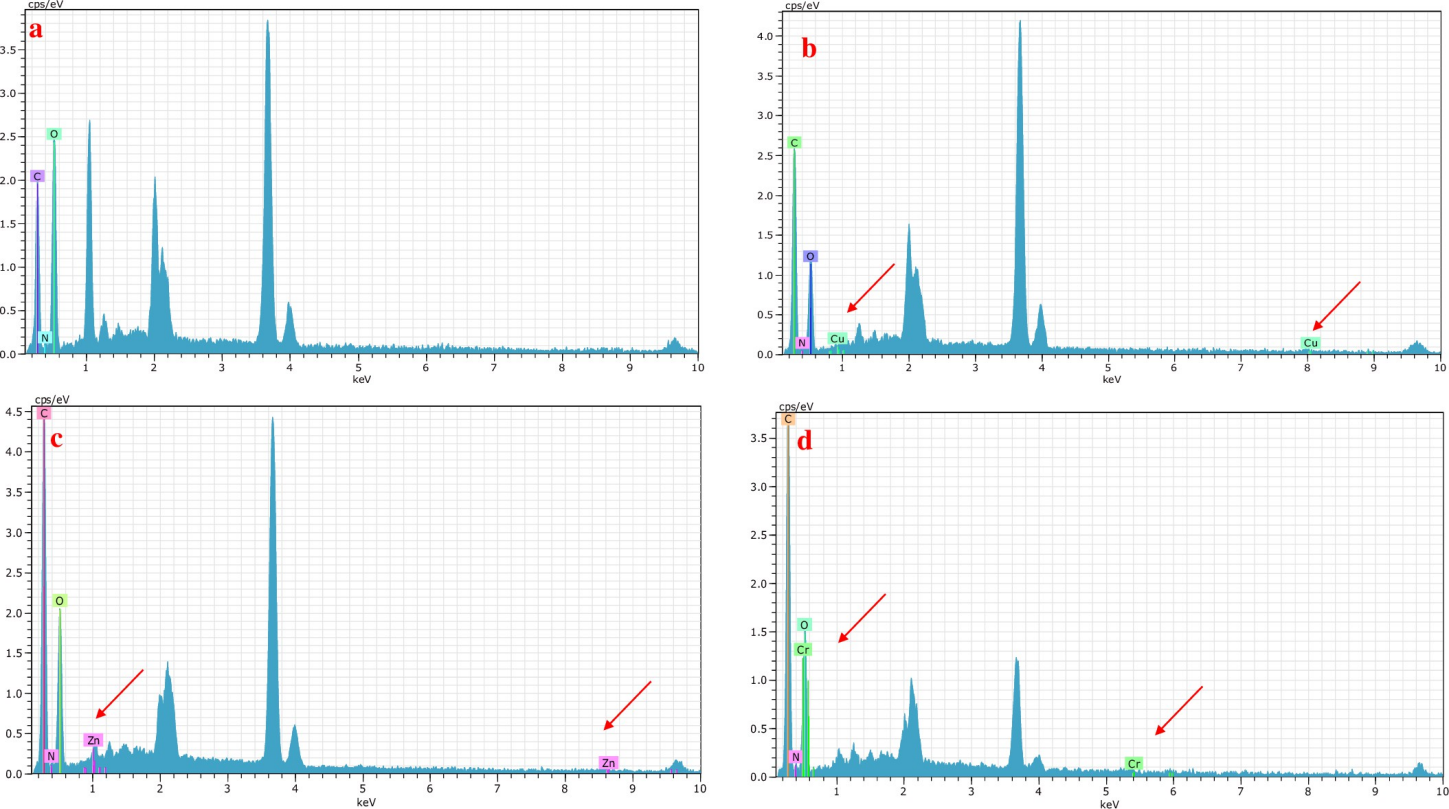
(a) EDX images of Imine before adsorption, (b-d) after adsorption of Cu(II), Zn(II), and Cr(III) onto Imine.

EDX result shows several compounds contained an imine- Schiff base **Fig 4A** Carbon and oxygen are the most dominant compounds. According to **Fig 4B**–**4D,** there is much Cu(II), Zn(II), and Cr(III) respectively present in the EDX spectrum on the adsorbent surface. This confirmed that the adsorption of these elements on the surface of the biomaterial was successful done.

#### XRD

**Fig 5:**
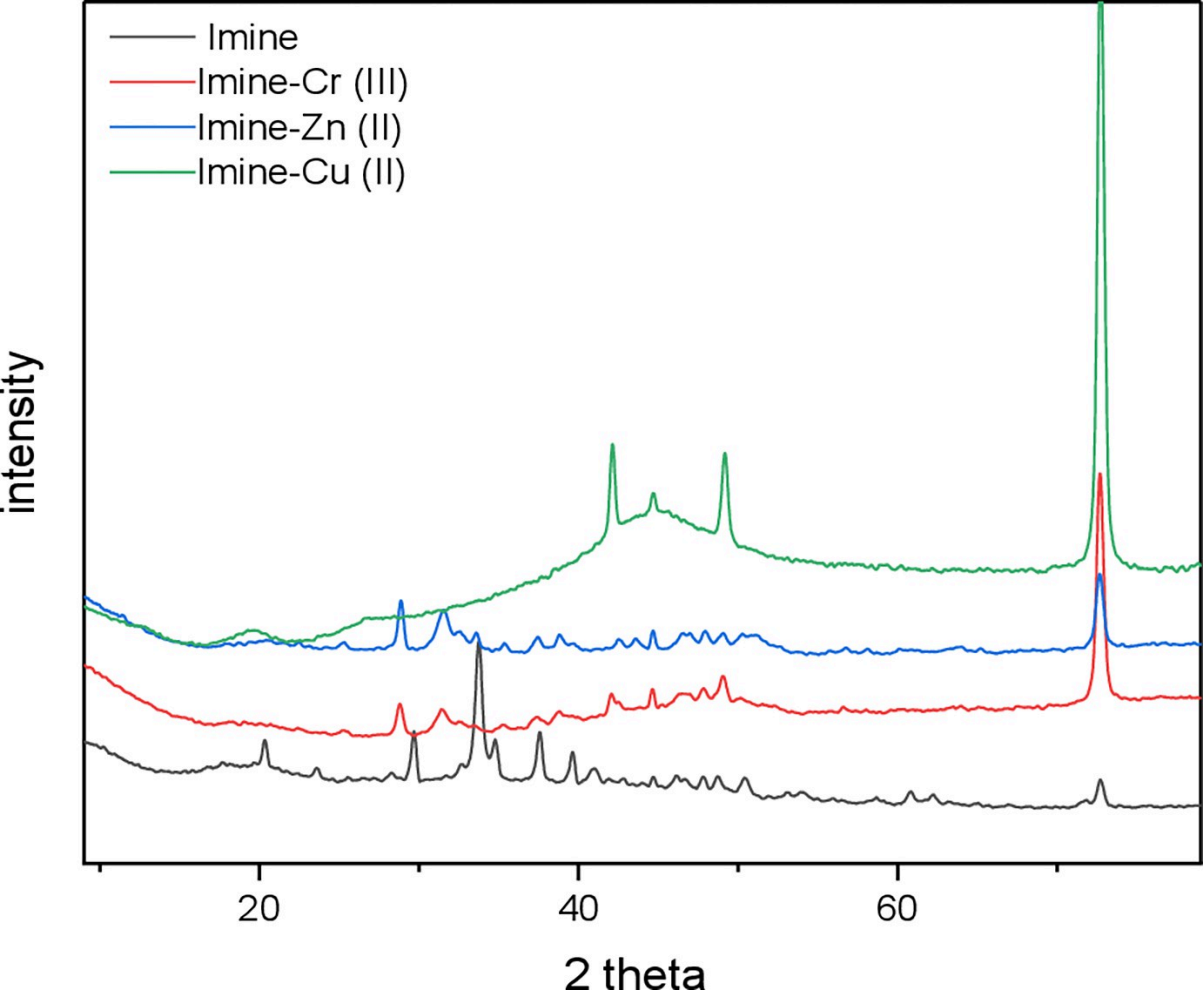
XRD spectra of Imine before and after Cu(II), Zn(II), and Cr(III) adsorption.

The crystallinity of the adsorbent is shown by X-ray diffraction; well-defined peaks reflect the material’s crystalline character, while the hallowed peak represents the material’s non-crystalline amorphous character. XRD patterns of Imine, Imine–Cu(II) ion, Imine–Zn(II) ion, Imine–Cr(III) ion are presented in **Fig 5**. When comparing the XRD patterns of imine loaded with Cu(II), Zn(II), and Cr(III) ions to that of the unloaded imine-Schiff base, it is discovered that the XRD pattern of imine loaded has significantly changed with a decrease in a crystalline structure. Consequently, it seems that the heavy metal ions preferentially adsorb via chemisorption and only partly through physisorption [24].

### Adsorption time–kinetic study

**Fig 6:**
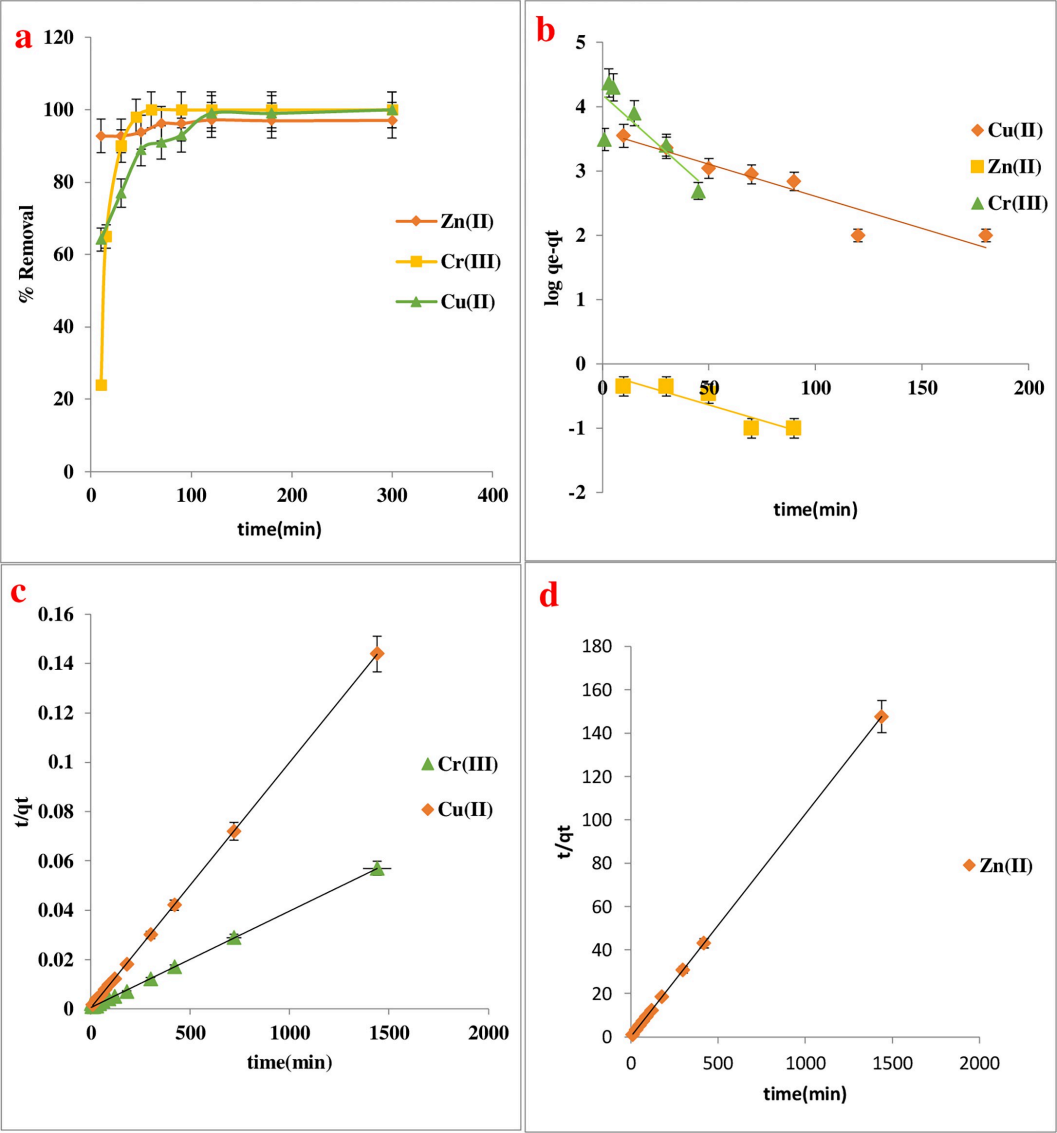
(a) The effect of contact time on the adsorption of Cu(II), Zn(II), and Cr(III) by Imine, (b)Pseudo-1^st^ order adsorption onto Imine, (c) Pseudo-2^nd^ order of Cu(II) and Cr(III) onto Imine, and (d) Pseudo-2^nd^ order for Zn(II) onto Imine.

It was decided to study the adsorption kinetics studies for Cu(II), Zn(II), and Cr(III) on a Schiff base were conducted with an initial concentration of 20 mg/L for Cu(II) ion and 50 mg/L for Zn(II) and Cr(III). The adsorbent dosage of 0.02 g, pH = 8 for Cu(II) ion and pH = 6 for Zn(II) and Cr(III) and 35°C. The findings are given in **Fig 6A.** It appears that the adsorption Cu(II)-imine ratio at the first 10 min showed 65%, then increased to 99% during 90 min due to more active sites for adsorption, and showed no significant change from 100 to 300 min because of occupation of the available adsorption sites. However, the zinc ion sees the light from first 10 min 93%, became 96.3% after 70 min, and then maintained equilibrium. While the adsorption rate was low during the first quarter of an hour for the Cr(III) ion, it rose after half-hour, and reached equilibrium after 45 min with an adsorption ratio of 100% [25]. This is attributed to the availability of large surface areas of the adsorbent. At these points, the equilibrium times were attained. After the plateau, the surface pores of the adsorbent became enclosed and reach the maximum uptake capacity. After that point the uptake rate be slow down of adsorption at this stage may be due to the agglomeration of metal ions on the surface of the adsorbent [26]. Two kinetic models of pseudo-1^st^ order and pseudo-2^nd^ order were used to investigate the process’s mechanism **Fig 6B–6D**. The details of the calculation rate constant (k), adsorption capacity, and correlation coefficient (R^2^) are shown in **Table 3** below. The calculation equilibrium adsorption capacity of Cu(II), Zn(II), and Cr(III) from the pseudo 2^nd^ order model (10 mg/g, 9.7 mg/g, and 25 mg/g) were close to the experiment qe (9.9 mg/g, 9.7 mg/g, and 24.9 mg/g). This demonstrated that this model may be used to estimate adsorption kinetics and the overall chemisorption has been a predominate mechanism by sharing or electron exchange between the imine surface and adsorbate ions [27].

**Table 3:** Kinetic study parameters of Cu(II), Cr(III) and Zn(II) ion adsorption of imine.

Adsorbents	qe (mg/g)	Pseudo 1^st^ order kinetic parameter			Pseudo 2^nd^ order kinetic parameter		
		q_cal_ (mg/g)	K_1_ (min ^-1^)	R^2^	q_cal_ (mg/g)	K_2_ (g/mg.min)	R^2^
**Imine-Cu(II)**	9.9	4.02	2.3	0.8964	10	0.02	0.9999
**Imine-Zn(II)**	9.7	1.39	0.0225	0.8276	9.7	0.015	0.9999
**Imine-Cr(III)**	24.9	15.1	2.95	0.6758	25	0.032	0.9991

### Effect of adsorption conditions

#### pH effects

The impact of pH is investigated to identify the adsorption pH at which maximal metal removal occurs. Batch adsorption was evaluated with the initial concentration of 20 mg/L for Cu(II) ion and 50 mg/L for Zn(II) and Cr(III). The adsorbent dosage of 0.02 g and 35°C. Hydrochloric acid or sodium hydroxide was used to adjust the pH from 3 to 11.

**Fig 7:**
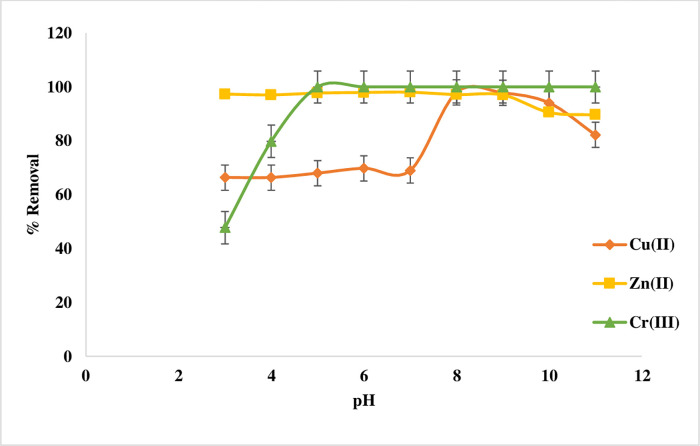
Curve of pH Effect for Cu(II), Zn(II), and Cr(III) adsorption on Imine.

The highest adsorption affinities for imine towards Cu(II) ion occur at pH 8 while pH 6 for Zn(II). At low pH less metal ion uptakes is observed due to the competitive adsorption H^+^ and metal ions. While the results show high adsorption ratio for Cr(III) in a wide range between 5–11 with 100% **Fig 7**. The reason of this trend is that hydrogen ions compete with metallic ions for active sites on the surface of the natural sorbent; this behaviour is anticipated. Because a tiny quantity of metal cations began to deposit as hydroxides at pH >9, metal ion retention became virtually steady [2,28]. The pH value of 8 was selected for Cu(II) as the optimal pH for this chelation [1,2] and pH 6 for Zn(II) and Cr(III) ions [29,30].

#### Metal concentration effects

**Fig 8:**
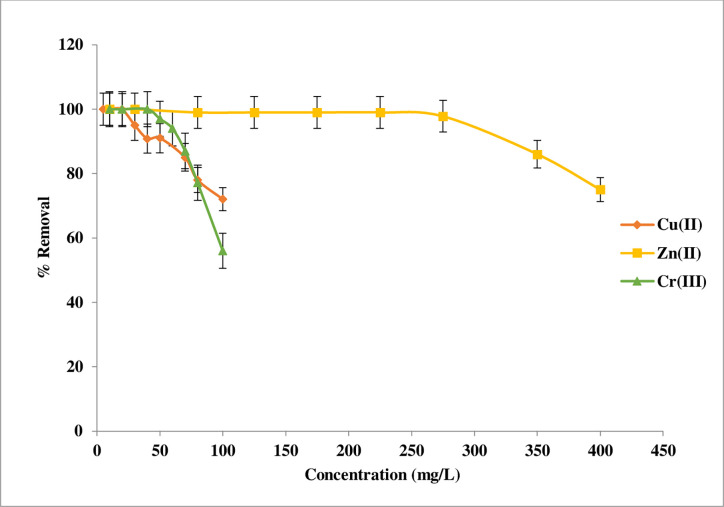
Curve of initial concentration Effect for Cu(II), Zn(II), and Cr(III) adsorption on Imine.

The effect of metal ion concentration on the adsorption parameters is a critical element to consider when applying an adsorbent in reality. This factor investigated under pH = 8 for Cu(II) ion and pH = 6 for Zn(II) and Cr(III) with 0.02g and 35°C. The variation in removal uptake and adsorption capacity towards Cu(II), Zn(II), and Cr(III) ions was seen in the current study as a function of metal ion concentration, which was varied in the experiment-from 10 to 100 mg/L for copper and chromium ion and from 10 to 400 mg/L for zinc ion. The reason for the different concentrations of zinc ions is that the adsorption ratio remains 100% within this limit. The results are presented in **Fig 8** It can be observed that when metal ion concentrations were raised, the removal uptake declined steadily. For explain this tendency, at lower concentrations, the ratios of available binding sites to the initial metal ion concentrations were larger, while at higher concentrations, the saturation of the adsorption sites occurred. This behaviour is attributed to less availability of surface-active sites [2,26].

#### Sorbent dose effects

**Fig 9:**
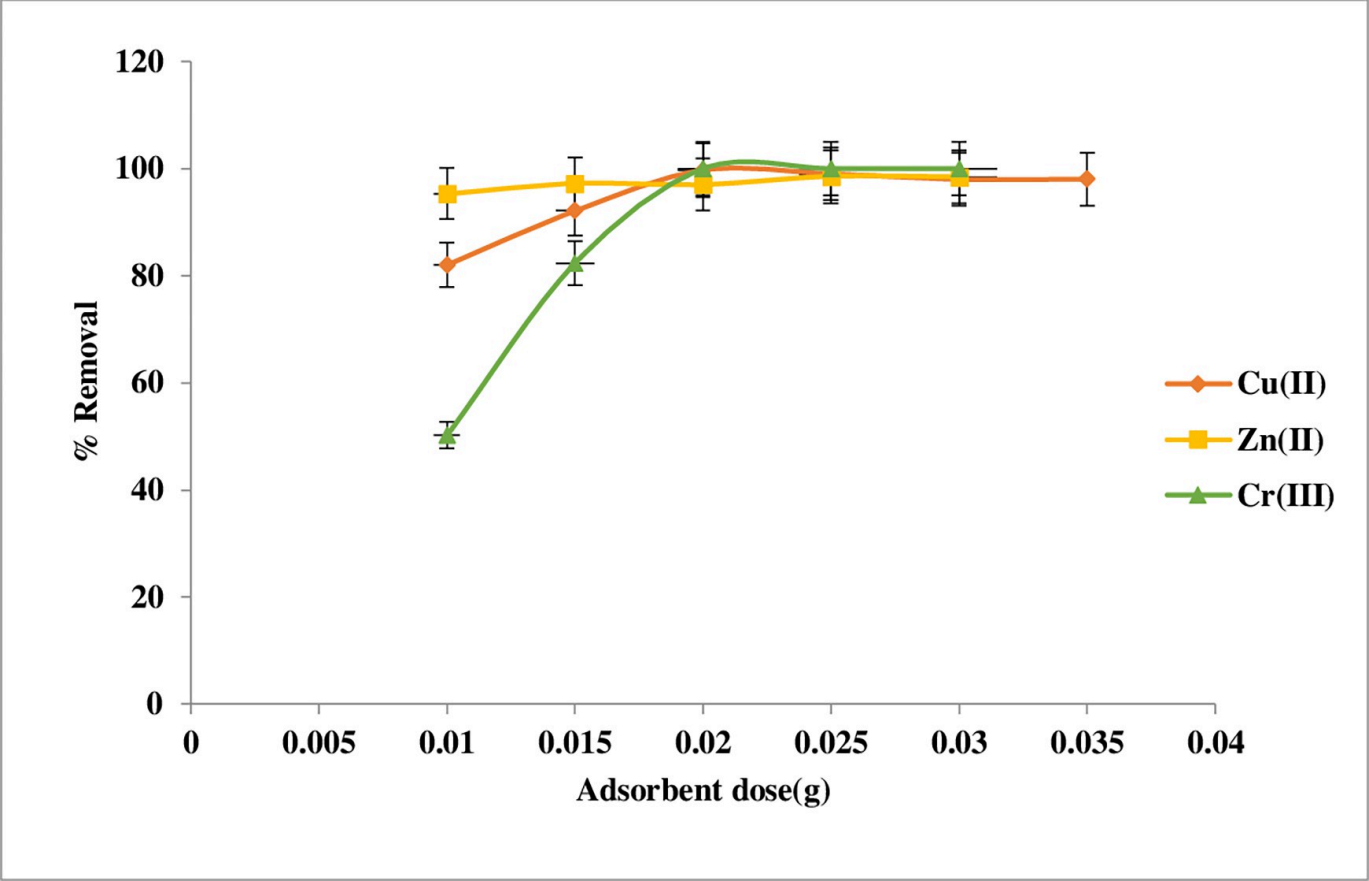
The effect of adsorbent dosage on the adsorption Cu(II), Zn(II), and Cr(III) by Imine.

The action of Schiff weight on heavy metal ions sorption was studied. As can be observed in **Fig 9** the optimal weight for adsorbent was 0.02 g, which resulted in 99.7%, 97%, and 100% removal for Cu(II), Zn(II), and Cr(III) respectively. Initially, the adsorption process increases as the adsorbent mass increases, but after an optimal dose is achieved, it stays constant. Due to a greater number of surface area, pore size and volume, and the availability of vacant sites, clearance efficiency is anticipated to rise as a consequence. Any further addition of the adsorbent seemed to have no significant effect on adsorption, which might be due to adsorption site overlapping because of adsorbent particle crowding [9,10]. Generally, 0.02 g was taken as an optimum quantity for this work.

#### Temperature effects

The temperature affects the mobility of molecules and ions in a solution. This may be extended to ion adsorption since ions must be mobile to ’collide/interact’ with the adsorbent and enhance adsorption, which is especially important in batch adsorption investigations [31]. Cu(II) ion shows high removal ratio at 35°C, which can be decreased with an increase in temperature. The decrease on the uptake of heavy metal ions with increase in temperature may be explained as a result of increase in the average kinetic energy of the metal ions; thus, making the attractive force between metal ions and polymer insufficient to retain the metal ions at the binding site. This could lead to desorption or cause the metal ions to bounce off the surface of the polymer instead of colliding and combining with it [32]. Surprisingly, within this small temperature range, the sorption capacities hardly change with increasing temperature; Cr(III) ion at all temperatures had the highest removal percentage of up to 100%, and changing temperature had no effect on the ratio of adsorption of Cr(III) Similar findings have been reported by other workers Benettayeb A. et al [33] as well as Zn(II) ion.

**Fig 10:**
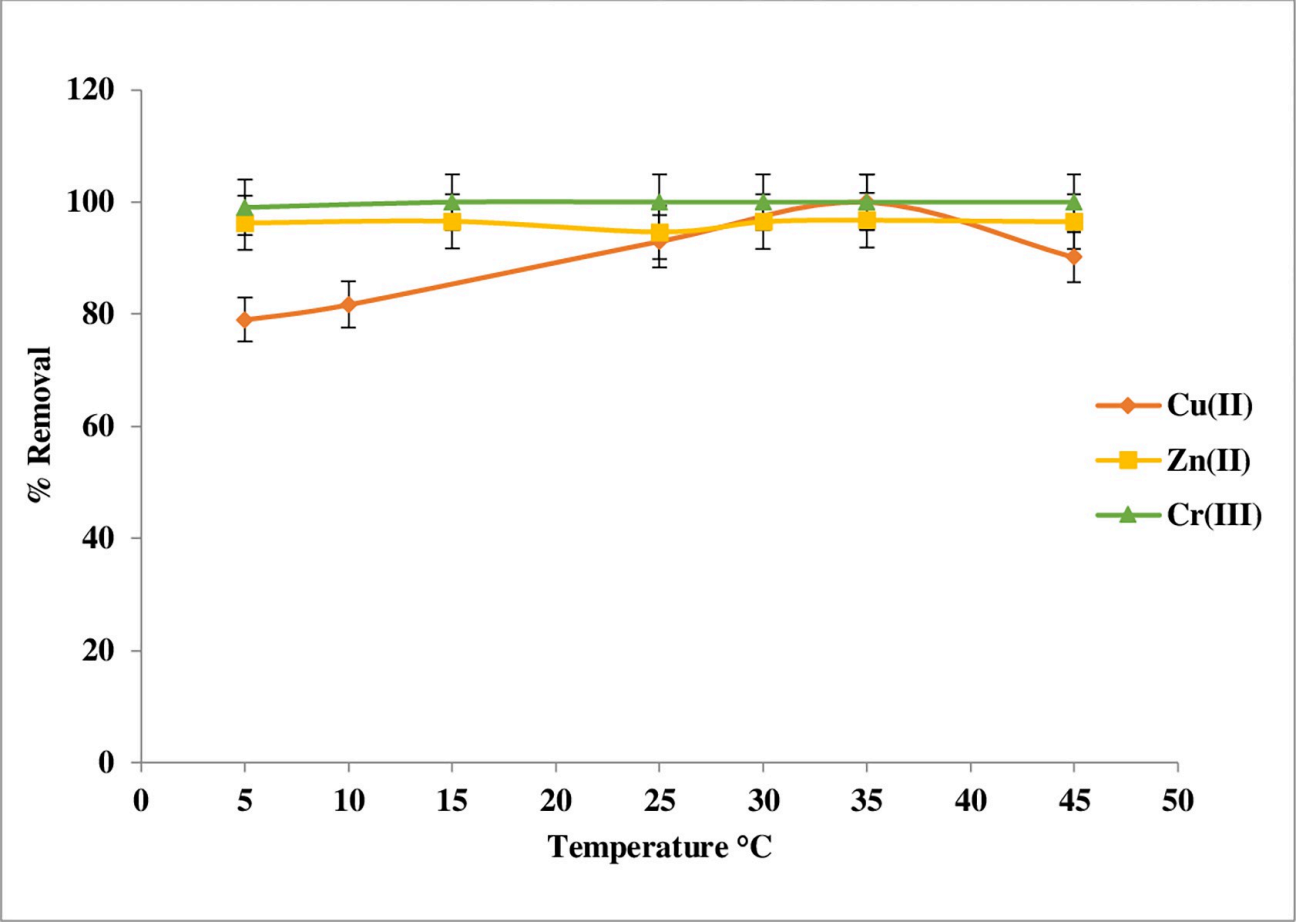
Temperature effect of Cu(II), Zn(II), and Cr(III) adsorption on Imine.

On the other hand, the Zn(II) ion had a 96% ratio of adsorption at low temperature which decreased to 94% when the temperature was increased to 25°C; the ratio then increased to 96% again when the temperature was higher than 35°C **Fig 10**. However, the variation observed between 5 and 45°C gives a first indication of the negligible impact of temperature on sorption performance [33].

Cu(II), Zn(II), and Cr(III) adsorption on Schiff base was examined at a temperature between 278 and 318 K. With the help of the following equations, we can determine the free energy change G (kJ/mol), the enthalpy change H (kJ/mol), and the entropy change s (kJ/mol K) of a system [34]:

$$Kd=\frac{qe}{Ce}$$ (8)

$$lnKd=\frac{\Delta S}{R}+\frac{\Delta H}{RT}$$ (9)

ΔG=‐RTlnKd (10)

where R denotes the universal gas constant (8.314 J/mol K), T denotes the absolute temperature (K), and Kd is the adsorption distribution constant deduced from qe/Ce (mg/g). The slope and intercept of the linear plot of ln Kd versus 1/T are used to get the values of ΔH and ΔS. In contrast, the values of ΔG were determined using the equation above [35]. As can be seen, the adsorption of Cu(II), Zn(II), and Cr(III) onto imine became more favorable as the temperature increased, suggesting an endothermic adsorption mechanism as seen by the positive values of ΔH **Table 4** The results indicate that chemisorption may be predominant. Following that, a positive value of ΔS implies a high degree of randomness at the interface between the solid and the solution. The negative values of ΔG indicate that the adsorption of heavy metal ions onto biosorbent occurred as a result of spontaneous adsorption [36].

**Table 4:** Thermodynamic parameters study for the adsorption of Cu(II), Zn(II) and Cr(III) ion adsorption on Imine.

Metal ion	T(K)	ΔG (kJ/mol)	ΔH(kJ/mol)	ΔS (J/mol k)
**Cu(II)**	283	-2.03	+ 133.01	+ 468.98
	298	-4.69		
	308	-1.59		
	318	-4.22		
**Zn(II)**	278	-5.91	+21.098	+25.5
	288	-6.34		
	298	-6.35		
	303	-6.6		
	308	-6.9		
	318	-6.92		
**Cr(III)**	278	-9.01	+69.72	+298.2
	288	-20.35		
	298	-21.08		
	308	-21.78		
	318	-22.47		

### Isotherm models’ study

**Fig 11:**
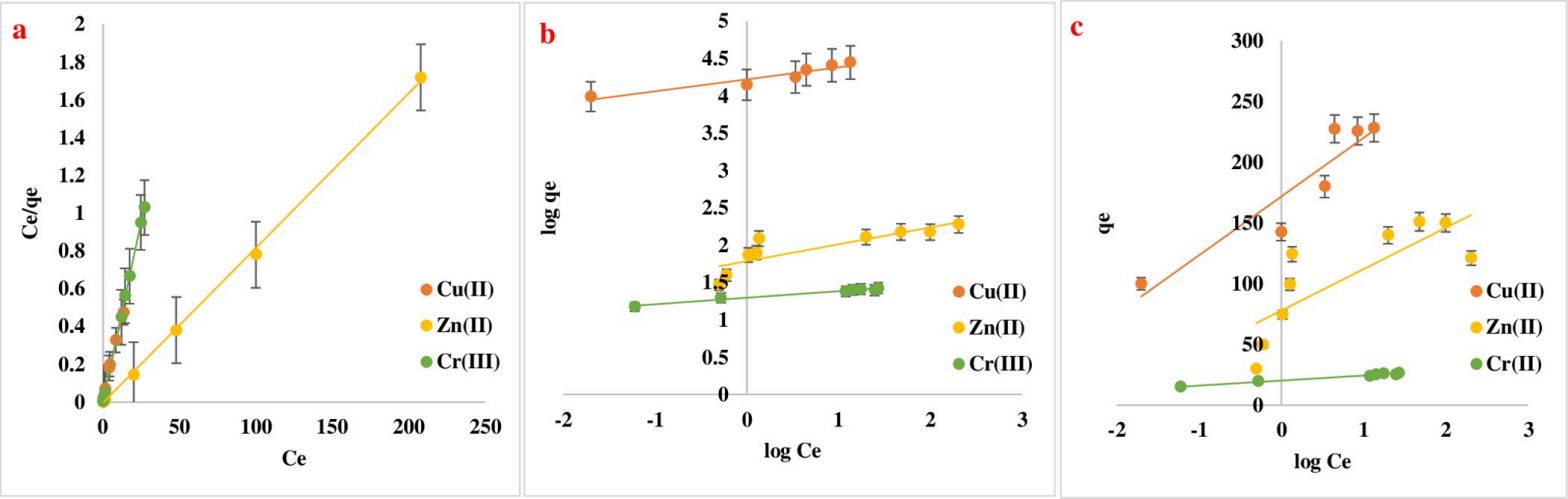
Adsorption isotherm of Cu(II), Zn(II) and Cr(III) onto Imine (a) linear plot of Langmuir model, (b) linear plot of Freundlich isotherm model, and (c) linear plot of Temkin Isotherm model.

The equilibrium adsorption isotherm is critical for characterizing the relationship between the solution and the adsorbent and building an adsorption system [37]. Isotherm investigations were carried out in batch mode, and the data were analyzed using three equilibrium models: Langmuir, Freundlich, and Temkin **Fig 11** The constants of the isotherms, which are determined from the slope and intercept of Eq (2) and Eq (3) for Langmuir, Eq (4) for Freundlich, and Eq (5) for Temkin are presented in **Table 5** As shown by the regression coefficients, the experimental data was well-fitting. In the Langmuir, R^2^ was 0.9998, 0.9991, and 0.9996 for Cu(II), Zn(II), and Cr(III) respectively. The Langmuir isotherm suggests that the adsorption mechanism was monolayer adsorption. High adsorption ability can an indication that the solute and adsorbent have a higher affinity. Another possible explanation is that the active sites are dealt with in an equal distribution on the surface and inside the adsorbent [19,27,35]. The separation factor R_L_ is favorable towards imine Cu(II), Zn(II) and Cr(III) ions with ranges (0.185–0.0434), (0.240–0.0069), and (0.0035–0.0013) respectively.

**Table 5:** Study of isotherm models correlation coefficients and constant for adsorption of Cu(II), Zn(II), and Cr(III) ion on imine.

Metal ion	Langmuir		Freundlich		Temkin	
**Cu(II)**	q_max_ (mg/g)	25	1/n	0.1603	at	171.59
	Kʟ	0.44	K*f*	16.81	bt	20.98
	R^2^	0.9998	R^2^	0. 9058	R^2^	0.8653
	Rʟ	(0.185–0.0434)				
**Zn(II)**	q_max_ (mg/g)	121	1/n	0.2276	at	7.8
	Kʟ	0.315	K*f*	61.1	bt	14.76
	R^2^	0.9991	R^2^	0.7159	R^2^	0.6296
	Rʟ	(0.240–0.0069)				
**Cr(III)**	q_max_ (mg/g)	26.31	1/n	0.0872	at	20.313
	Kʟ	9.26	*f*	19.77	bt	1.775
	R^2^	0.9996	R^2^	0.9737	R^2^	0.9756
	Rʟ	(0.0035–0.0013)				

### Desorption and reuse

The capacity to be reabsorbed and reused is one of the main characteristics that make an adsorbent useful and essential for improving process economics. The Schiff base adsorbents have Cu(II), Zn(II), and Cr(III) adsorbed on their surface. The copper and zinc ions will be next dehydrated and treated with 0.1 M EDTA solutions while 0.1M EDTA with 1M HCl (1:1) will be used for chromium ion.

**Fig 12:**
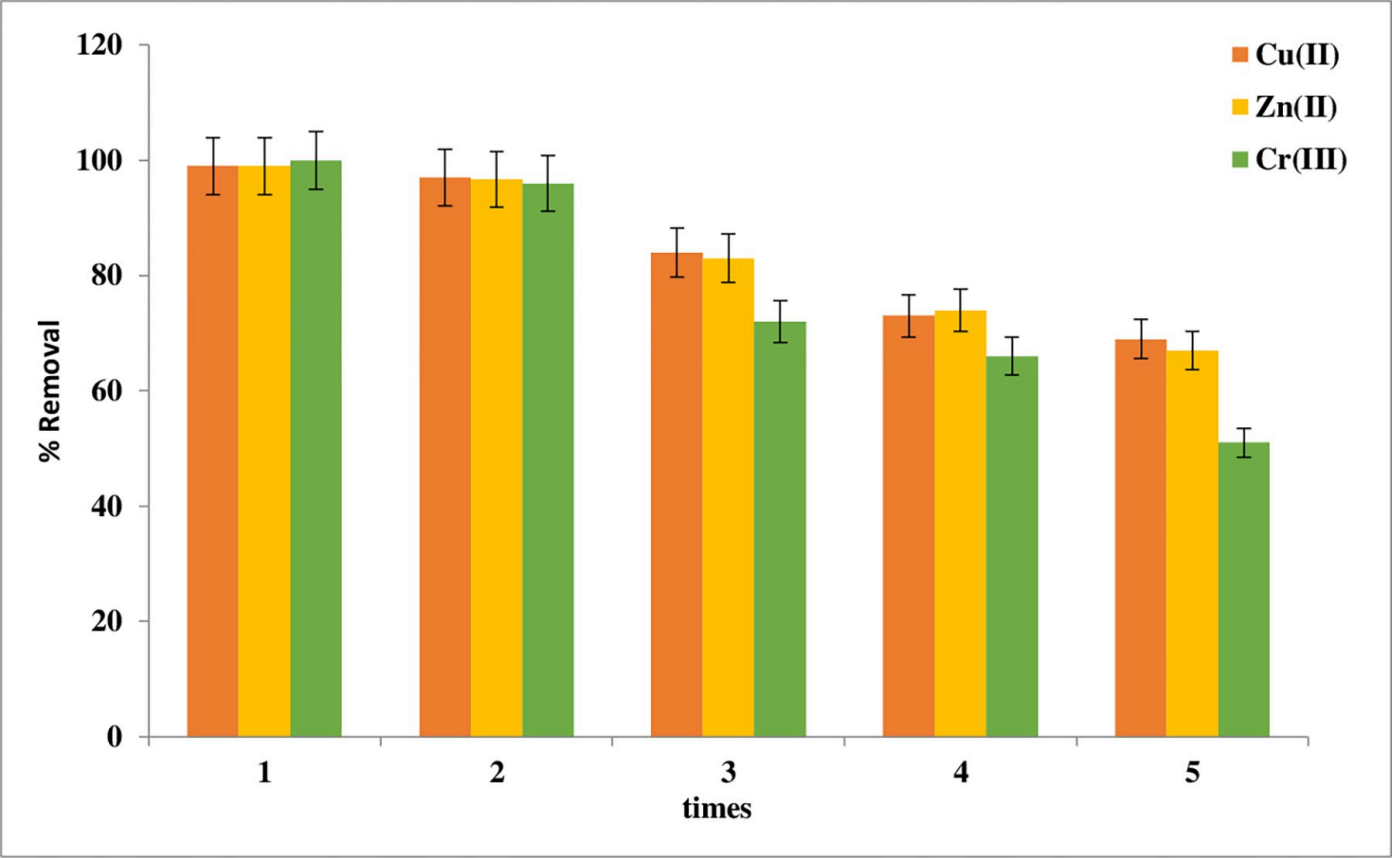
Reusability of imine for removal percent to Cu(II), Zn(II), and Cr(III).

The adsorption/desorption tests are then repeated five times for Schiff base adsorption-desorption periods, as shown in **Fig 12** When used five times, the efficiency of imine was significantly reduced after the third cycle, falling to 84%, 83%, and 72% for Cu(II), Zn(II), and Cr(III) respectively. Furthermore, the capacity of the Schiff base had decreased to less than 67% for Cu(II) and Zn(II) after the fifth period while it was less than 51% for Cr(III) ion [11,38,39].

#### Comparison with another study

The adsorption capacity of the imine used for removal of the Cu(II), Zn(II), and Cr(III) ions was compared to those of other adsorbent materials cited in the literature. The data compiled in **Table 6** show the adsorption capacities obtained from the Langmuir model.

**Table 6:** Comparison of Cu(II), Zn(II) and, Cr(III) adsorbent in different adsorbents materials.

Adsorbents	Metal ion	Adsorption capacity (qmax) (mg/g)	Reference
**Poly (2-hydroxyethyl methacrylate-n-vinyl imidazole) [poly(HEMA-VIM)] -cryogel**	Cu(II) Zn(II)	2.5 4.340	[40]
**(SG-H_2_L) ditopic -zwitterionic Schiff base ligand H_2_L 1 onto a modified silica gel**	Cu(II)	41.31	[41]
**5-methyl-2-thiophenecarboxaldehyde Schiff base-immobilised -SBA-15**	Cr(III) Zn(II)	37 32	[42]
**Fe_3_O_4_@SiO_2_/Schiff base**	Cu(II) Zn(II)	9.2 87	[30]
**chitosan/attapulgite composites (CTS/ATP)**	Cr(III)	27.03	[43]
**Schiff base ligands 3-methoxy salicylaldimine propyl triethoxysilane (MNS1), 5-bromo salicylaldimine propyl triethoxysilane (MNS2)**	Cu(II)	3.61	[44]
**silicate–chitosan composite**	Cr(III)	0.89	[45]
**Chitosan–imine**	Cu(II) Zn(II) Cr(III)	25 121 26.31	This study

### Real sample application

To obtain controls for the study, shown in Table 7 samples were spiked with Cu(II), Zn(II), and Cr(III) ions to supplement tablets. After the batch experiment, the recovery of Cu(II), Zn(II), and Cr(III) ions in real and spiked samples varied from 97.84%–99.80%, 99.2%–99.7%, and 98.4%–99.9%, respectively. Relative stander deviation was less than 1.32 for copper ion, 0.76 for zinc ion, and 1.2 for chromium ion with imine. The results support the sensitivity and reliability of adsorbent toward spike and non-spike for preconcentration and determination of these three ions in trace value. Statistical analysis showed that there was a significant difference between the recovery of three ions Cu(II), Zn(II), and Cr(III) ions in real and spiked samples (p < 0.05).

**Table 7:** Determination of Cu(II), Zn(II), and Cr(III) ion in two supplements.

Adsorbent	Samples	Amount added μg/mL	Amount found μg/mL	RSD	Recovery %
**Cu(II)**	Heavy metal supplement	0 30 50	15 44.9 64.7	± 1.2 ± 0.30 ± 0.38	- 99.77 99.5
	Multivitamine Supplement	0 30 50	1 30.94 49.90	0 ± 0.92 ± 1.32	- 99.80 97.84
**Zn(II)**	Heavy metal supplement	0 30 50	70 99.97 119.07	0 ±0.76 0.54	- 99.7 99.2
	Multivitamine Supplement	0 30 50	2 31.84 51.79	0 ±0.001 ±0.1	- 99.5 99.59
**Cr(III)**	Heavy metal supplement	0 30 50	5 34.79 54.97	± 1.2 ± 0.30 ± 0.38	- 99.4 99.9
	Multivitamine Supplement	0 30 50	0.5 30.24 49.74	0 ± 0.92 ± 1.012	- 99.1 98. 4

### Statistical analysis

IBM SPSS statistics (V. 23). T-Test was applied to analyze the collected data. A P-value of < 0.05 was considered statistically significant.

## Conclusion

Chitosan was effectively and covalently functionalized on the 2,2’-[propane-1,3-diylbis(oxy)] dibenzaldehyde surface. A variety of spectroscopic methods were used to investigate the physico-chemical properties of the compound. Imine with a large surface area and small volume removes metal ions Cu(II), Zn(III), and Cr(III) efficiently and swiftly. The approach of continuous adsorption is the most cost-effective. Imine-Schiff base removal of heavy metal ions is an innovative, quick, and cost-effective method based on the graphs and tables presented. The adsorption kinetics of heavy metals on adsorbent followed a pseudo-2^nd^ order model. The equilibrium findings agree well with the Langmuir adsorption model, indicating monolayer coverage of heavy metal molecules at the imine-Schiff base’s outer surface where Cu(II), Zn(II), and Cr(III) had a maximum sorption capacity, q_max_, of 25 mg/g, 121 mg/g, and 26,31 mg/g respectively, according to the results. This heavy metal ion adsorption technique is a dependable, less hazardous, cost-effective, and time-effective novel approach.

## Supporting information

S1 Fig Represents 1H-NMR spectrum 2,2’-(propane-1,3-diylbis(oxy)) dibenzaldehyde (compound 1). (DOCX)

S2 Fig H-NMR for imine (2-(3-(2-((E)-(((2R,3R,4R,5S,6S)-4-hydroxy-6-(hydroxymethyl)-2-methoxy-5-methyltetrahydro-2H-pyryl) imino) methyl) phenoxy) propoxy) benzaldehyde. (DOCX)

## References

[1] Bhargava S, Uma V. Rapid extraction of Cu (II) heavy metal from industrial waste water by using silver nanoparticles anchored with novel Schiff base. Separation Science and Technology. 2019;54(7):1182–93.

[2] Hassan R, Arida H, Montasser M, Abdel Latif N. Synthesis of new Schiff base from natural products for remediation of water pollution with heavy metals in industrial areas. Journal of Chemistry. 2013;2013.

[3] Zhou Z, Kong D, Zhu H, Wang N, Wang Z, Wang Q, et al. Preparation and adsorption characteristics of an ion-imprinted polymer for fast removal of Ni (II) ions from aqueous solution. Journal of hazardous materials. 2018;341:355–64. doi: 10.1016/j.jhazmat.2017.06.010 28802246

[4] Hamza MF, Gamal A, Hussein G, Nagar MS, Abdel‐Rahman AAH, Wei Y, et al. Uranium (VI) and zirconium (IV) sorption on magnetic chitosan derivatives–effect of different functional groups on separation properties. Journal of Chemical Technology & Biotechnology. 2019;94(12):3866–82.

[5] Chen N, Kong P, Feng H, Wang Y, Bai D. Corrosion Mitigation of Chitosan Schiff Base for Q235 Steel in 1.0 M HCl. Journal of Bio-and Tribo-Corrosion. 2019;5(1):27.

[6] Triana-Guzmán VL, Ruiz-Cruz Y, Romero-Peñaloza EL, Zuluaga-Corrales HF, Chaur-Valencia MN. New chitosan-imine derivatives: from green chemistry to removal of heavy metals from water. Revista Facultad de Ingeniería Universidad de Antioquia. 2018(89):34–43.

[7] Zhao H, Caldora HP, Turner O, Douglas JJ, Leonori D. A Desaturative Approach for Aromatic Aldehyde Synthesis via Synergistic Enamine, Photoredox and Cobalt Triple Catalysis. Angewandte Chemie. 2022;134(18):e202201870. doi: 10.1002/anie.202201870 35196413 PMC9311220

[8] Abu-Yamin A-A, Abduh MS, Saghir SAM, Al-Gabri N. Synthesis, Characterization and Biological Activities of New Schiff Base Compound and Its Lanthanide Complexes. Pharmaceuticals. 2022;15(4):454. doi: 10.3390/ph15040454 35455451 PMC9027428

[9] Gokila S, Gomathi T, Sudha P, Anil S. Removal of the heavy metal ion chromiuim (VI) using Chitosan and Alginate nanocomposites. International journal of biological macromolecules. 2017;104:1459–68. doi: 10.1016/j.ijbiomac.2017.05.117 28551438

[10] Nematidil N, Sadeghi M, Nezami S, Sadeghi H. Synthesis and characterization of Schiff-base based chitosan-g-glutaraldehyde/NaMMTNPs-APTES for removal Pb2+ and Hg2+ ions. Carbohydrate polymers. 2019;222:114971. doi: 10.1016/j.carbpol.2019.114971 31320055

[11] Shahraki S, Delarami HS, Khosravi F, Nejat R. Improving the adsorption potential of chitosan for heavy metal ions using aromatic ring-rich derivatives. Journal of colloid and interface science. 2020;576:79–89. doi: 10.1016/j.jcis.2020.05.006 32413783

[12] Kocak N, Sahin M, Kücükkolbasi S, Erdogan ZO. Synthesis and characterization of novel nano-chitosan Schiff base and use of lead (II) sensor. International journal of biological macromolecules. 2012;51(5):1159–66. doi: 10.1016/j.ijbiomac.2012.09.003 22982811

[13] Ahmed MO, Shrpip A, Mansoor M. Synthesis and characterization of new schiff base/thiol-functionalized mesoporous silica: an efficient sorbent for the removal of Pb (II) from aqueous solutions. Processes. 2020;8(2):246.

[14] Puvvada YS, Vankayalapati S, Sukhavasi S. Extraction of chitin from chitosan from exoskeleton of shrimp for application in the pharmaceutical industry. International Current Pharmaceutical Journal. 2012;1(9):258–63.

[15] De Queiroz Antonino RSCM, Lia Fook BRP, de Oliveira Lima VA, de Farias Rached RÍ, Lima EPN, da Silva Lima RJ, et al. Preparation and characterization of chitosan obtained from shells of shrimp (Litopenaeus vannamei Boone). Marine drugs. 2017;15(5):141. 10.3390/md15050141 PMC5450547 28505132

[16] Ilhan S, Temel H, KIlIc A. Synthesis and spectral studies of macrocyclic Cu (II) complexes by reaction of various diamines, copper (II) perchlorate and 1, 4-bis (2-carboxyaldehyde phenoxy) butane. Journal of Coordination Chemistry. 2008;61(2):277–84.

[17] Vadivel T, Dhamodaran M, Kulathooran S, Kavitha S, Amirthaganesan K, Chandrasekaran S, et al. Rhodium (III) complexes derived from complexation of metal with azomethine linkage of chitosan biopolymer Schiff base ligand: Spectral, thermal, morphological and electrochemical studies. Carbohydrate research. 2020;487:107878. doi: 10.1016/j.carres.2019.107878 31760235

[18] Ayawei N, Ebelegi AN, Wankasi D. Modelling and interpretation of adsorption isotherms. Journal of chemistry. 2017;2017.

[19] Zhang Y, Bai Z, Luo W, Zhai L, Wang B, Kang X, et al. Ion imprinted adsorbent for the removal of Ni (II) from waste water: preparation, characterization, and adsorption. Journal of Dispersion Science and Technology. 2019;40(12):1751–60.

[20] Monier M, Bukhari AAH, Elsayed NH. Designing and characterization of copper (II) ion-imprinted adsorbent based on isatin functionalized chitosan. International journal of biological macromolecules. 2020;155:795–804. doi: 10.1016/j.ijbiomac.2020.03.215 32229208

[21] Kumaran JS, Priya S, Jayachandramani N, Mahalakshmi S. Synthesis, spectroscopic characterization and biological activities of transition metal complexes derived from a tridentate Schiff base. Journal of Chemistry. 2013;2013.

[22] Thatte C, Rathnam M, Pise A. Chitosan-based Schiff base-metal complexes (Mn, Cu, Co) as heterogeneous, new catalysts for the β-isophorone oxidation. Journal of chemical sciences. 2014;126(3):727–37.

[23] Issa Y, Hassib H, Abdelaal H. 1H NMR, 13C NMR and mass spectral studies of some Schiff bases derived from 3-amino-1, 2, 4-triazole. Spectrochimica Acta Part A: Molecular and Biomolecular Spectroscopy. 2009;74(4):902–10. 19783202 10.1016/j.saa.2009.08.042

[24] Elavarasan A. EDX and XRD, FT-IR spectra, analysis containing hexavalent chromium metal ion adsorption present in aqueous solution on to phosphoric acid (H_3_PO_4_) activated mimusops elengi leaves carbon. Journal of Drug Delivery and Therapeutics. 2018;8(5-s):132–8.

[25] Bayramoglu G, Arica MY. Synthesis of Cr (VI)-imprinted poly (4-vinyl pyridine-co-hydroxyethyl methacrylate) particles: its adsorption propensity to Cr (VI). Journal of Hazardous Materials. 2011;187(1–3):213–21. doi: 10.1016/j.jhazmat.2011.01.022 21272995

[26] Mustapha S, Shuaib D, Ndamitso M, Etsuyankpa M, Sumaila A, Mohammed U, et al. Adsorption isotherm, kinetic and thermodynamic studies for the removal of Pb (II), Cd (II), Zn (II) and Cu (II) ions from aqueous solutions using Albizia lebbeck pods. Applied water science. 2019;9(6):1–11.

[27] Ren Y, Zhang M, Zhao D. Synthesis and properties of magnetic Cu (II) ion imprinted composite adsorbent for selective removal of copper. Desalination. 2008;228(1–3):135–49.

[28] Vasconcelos HL, Guibal E, Laus R, Vitali L, Fávere VT. Competitive adsorption of Cu (II) and Cd (II) ions on spray-dried chitosan loaded with Reactive Orange 16. Materials Science and Engineering: C. 2009;29(2):613–8.

[29] Pakade V, Cukrowska E, Darkwa J, Torto N, Chimuka L. Selective removal of chromium (VI) from sulphates and other metal anions using an ion-imprinted polymer. Water Sa. 2011;37(4):529–38.

[30] Moradinasab S, Behzad M. Removal of heavy metals from aqueous solution using Fe3O4 nanoparticles coated with Schiff base ligand. Desalination and Water Treatment. 2016;57(9):4028–36.

[31] Mafu LD, Mamba BB, Msagati TA. Synthesis and characterization of ion imprinted polymeric adsorbents for the selective recognition and removal of arsenic and selenium in wastewater samples. Journal of Saudi Chemical Society. 2016;20(5):594–605.

[32] Abd El-Latif M, Ibrahim AM, El-Kady M. Adsorption equilibrium, kinetics and thermodynamics of methylene blue from aqueous solutions using biopolymer oak sawdust composite. Journal of American science. 2010;6(6):267–83.

[33] Benettayeb A, Morsli A, Elwakeel KZ, Hamza MF, Guibal E. Recovery of Heavy Metal Ions Using Magnetic Glycine-Modified Chitosan—Application to Aqueous Solutions and Tailing Leachate. Applied Sciences. 2021;11(18):8377.

[34] Zeng J, Chen H, Yuan X, Guo Q, Yu X. A ion-imprinted chitosan/Al_2_O_3_ composite material for selective separation of copper (II). Desalination and Water Treatment. 2015;55(5):1229–39.

[35] He Y, Wu P, Xiao W, Li G, Yi J, He Y, et al. Efficient removal of Pb (II) from aqueous solution by a novel ion imprinted magnetic biosorbent: Adsorption kinetics and mechanisms. PLoS One. 2019;14(3):e0213377. doi: 10.1371/journal.pone.0213377 30917141 PMC6437713

[36] Van Thuan Le MUD, Hoang Sinh Le, Dai Lam Tran, Van Dat Doan, Nguyen HT. Adsorption of Ni(II) ions by magnetic activated carbon/chitosan beads prepared from spent coffee grounds, shrimp shells and green tea extract. Environmental Technology. 2019(ISSN: 0959-3330): 10. doi: 10.1080/09593330.2019.1584250 30767655

[37] Hassanpour S, Taghizadeh M, Yamini Y. Magnetic Cr (VI) ion imprinted polymer for the fast selective adsorption of Cr (VI) from aqueous solution. Journal of Polymers and the Environment. 2018;26(1):101–15.

[38] Fan H-T, Sun X-T, Zhang Z-G, Li W-X. Selective removal of lead (II) from aqueous solution by an ion-imprinted silica sorbent functionalized with chelating N-donor atoms. Journal of Chemical & Engineering Data. 2014;59(6):2106–14.

[39] Xu X, Wang M, Wu Q, Xu Z, Tian X. Synthesis and application of novel magnetic ion-imprinted polymers for selective solid phase extraction of cadmium (II). Polymers. 2017;9(8):360. 10.3390/polym9080360 PMC6418836 30971037

[40] Tekin K, Uzun L, Şahin ÇA, Bektaş S, Denizli A. Preparation and characterization of composite cryogels containing imidazole group and use in heavy metal removal. Reactive and Functional Polymers. 2011;71(10):985–93.

[41] Wang Q, Gao W, Liu Y, Yuan J, Xu Z, Zeng Q, et al. Simultaneous adsorption of Cu (II) and SO42− ions by a novel silica gel functionalized with a ditopic zwitterionic Schiff base ligand. Chemical Engineering Journal. 2014;250:55–65.

[42] Parambadath S, Mathew A, Barnabas MJ, Kim SY, Ha C-S. Concentration-dependant selective removal of Cr (III), Pb (II) and Zn (II) from aqueous mixtures using 5-methyl-2-thiophenecarboxaldehyde Schiff base-immobilised SBA-15. Journal of Sol-Gel Science and Technology. 2016;79(3):426–39.

[43] Zou X, Pan J, Ou H, Wang X, Guan W, Li C, et al. Adsorptive removal of Cr (III) and Fe (III) from aqueous solution by chitosan/attapulgite composites: Equilibrium, thermodynamics and kinetics. Chemical Engineering Journal. 2011;167(1):112–21.

[44] Moftakhar MK, Yaftian M, Ghorbanloo M. Adsorption efficiency, thermodynamics and kinetics of Schiff base-modified nanoparticles for removal of heavy metals. International Journal of Environmental Science and Technology. 2016;13(7):1707–22.

[45] Copello G, Varela F, Vivot RM, Diaz L. Immobilized chitosan as biosorbent for the removal of Cd (II), Cr (III) and Cr (VI) from aqueous solutions. Bioresource technology. 2008;99(14):6538–44. doi: 10.1016/j.biortech.2007.11.055 18166453

