# Supplementary material for: Rapid adsorption of some heavy metals using extracted chitosan anchored with new aldehyde to form a schiff base
Source: PLoS One. 2022 Sep 9;17(9):e0274123. doi: 10.1371/journal.pone.0274123 (PMC9462815; doi:10.1371/journal.pone.0274123)
Supplement: S1 Fig — (DOCX) [file pone.0274123.s001.docx]

**
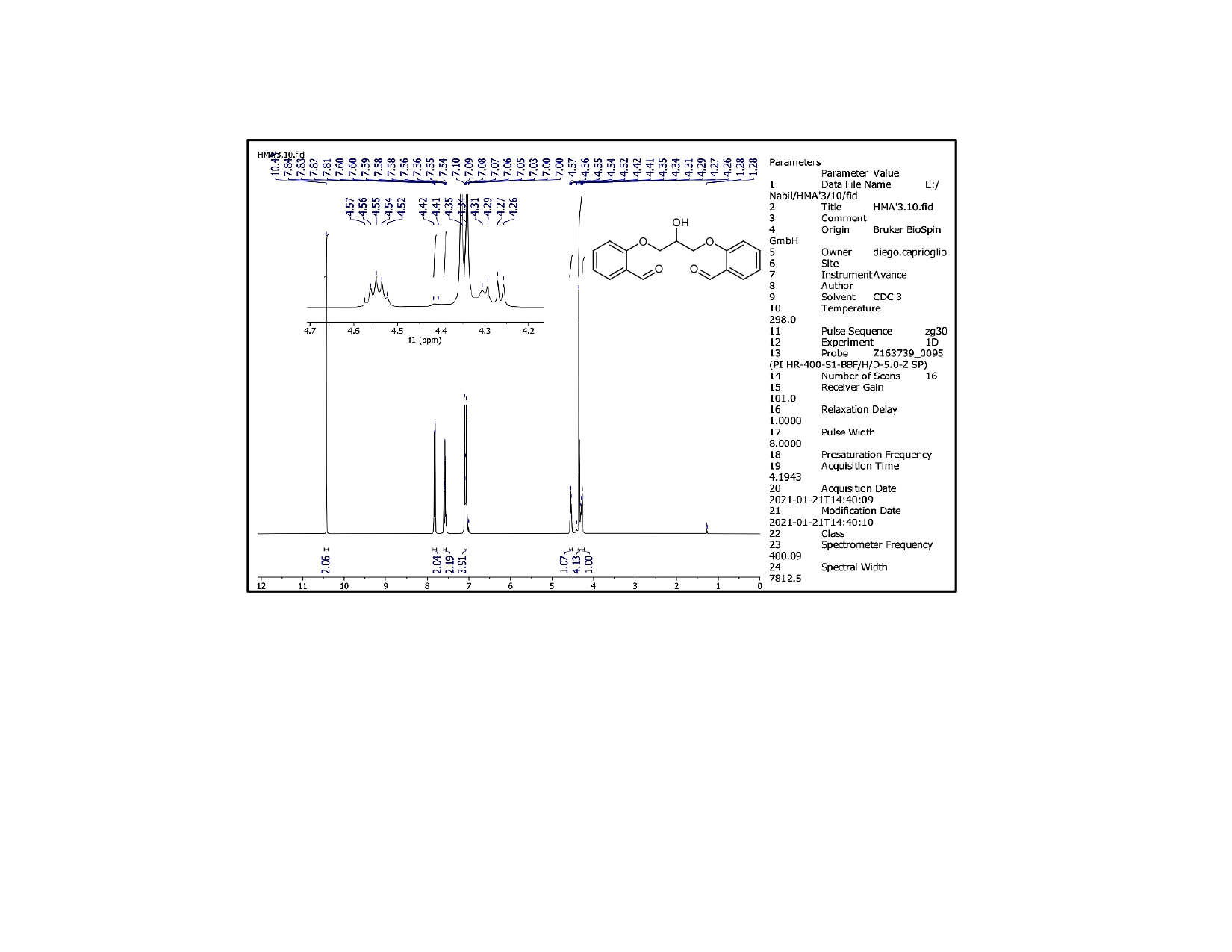
**

S1 Fig. Represents ^1^H-NMR spectrum 2,2'-(propane-1,3-diylbis(oxy)) dibenzaldehyde (compound 1)

**S1 Fig Represents ^1^H-NMR spectrum 2,2'-(propane-1,3-diylbis(oxy)) dibenzaldehyde (compound 1)**
