# Supplementary material for: Rapid adsorption of some heavy metals using extracted chitosan anchored with new aldehyde to form a schiff base
Source: PLoS One. 2022 Sep 9;17(9):e0274123. doi: 10.1371/journal.pone.0274123 (PMC9462815; doi:10.1371/journal.pone.0274123)
Supplement: S2 Fig — (DOCX) [file pone.0274123.s002.docx]

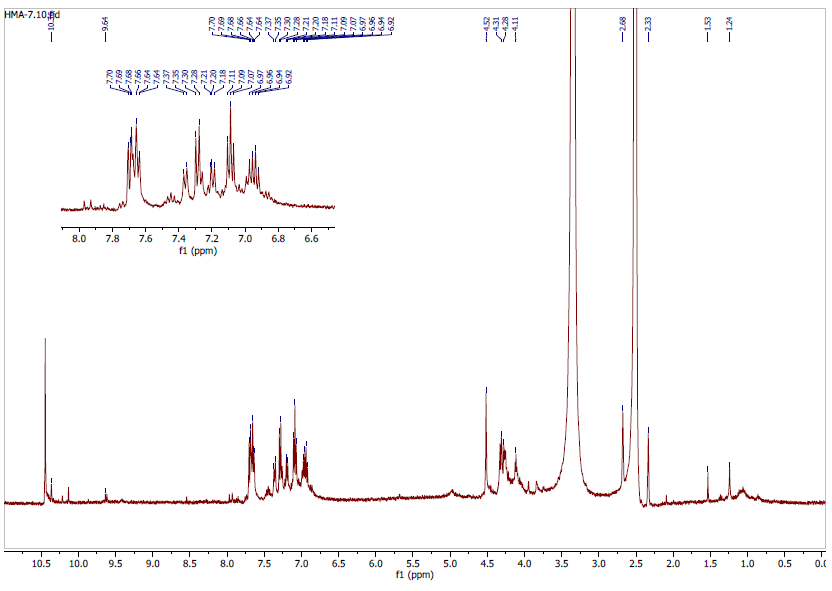


**S2 Fig** **.H-NMR for imine (2-(3-(2-((E)-(((2R,3R,4R,5S,6S)-4-hydroxy-6-(hydroxymethyl)-2-methoxy-5-methyltetrahydro-2H-pyryl) imino) methyl) phenoxy) propoxy) benzaldehyde**
